# Parallel addictions: understanding the psychological and neurobehavioral damage of gaming and alcohol use in adolescents and young adults

**DOI:** 10.3389/fpsyg.2026.1805339

**Published:** 2026-08-03

**Authors:** Abhijeet Singh, Sneha Sharma, Fayaz Ahmad Paul, V. Kalyani

**Affiliations:** 1 School of Behavioural Sciences and Forensic Investigations, Rashtriya Raksha University, Gandhinagar, India; 2 Prem Neuropsychiatry Centre, Malerkotla, India; 3 Department of Hospital Administration, All India Institute of Medical Sciences (AIIMS), Rajkot, India; 4 Department of Social Work, Rajagiri College of Social Sciences (Autonomous), Kochi, India

**Keywords:** adolescents, alcohol use disorder, emotional regulation, executive dysfunction, Gaming Disorder, neurobehavioral vulnerability

## Abstract

**Introduction:**

Adolescence and young adulthood are critical neurodevelopmental periods characterized by increased vulnerability to addictive behaviors due to ongoing maturation of reward-processing and executive-control systems. Gaming Disorder (GD) and Alcohol Use Disorder (AUD) have emerged as significant behavioral and public mental health concerns among youth populations. To review and synthesize the psychological, cognitive, psychosocial and neurobiological correlates of GD and AUD in adolescents and young adults and to consider their common and disorder-specific vulnerabilities.

**Methods:**

A narrative review was carried out following principles guided by SANRA. A structured literature search was conducted in PubMed, Scopus, PsycINFO and Google Scholar for peer-reviewed studies published between 2010 and 2025. Thirty-three studies fulfilling the inclusion criteria were thematically synthesized across neurobiological, emotional, cognitive and psychosocial domains.

**Results:**

Gaming Disorder (GD) and Alcohol Use Disorder (AUD) showed significant overlap in emotional dysregulation, impulsivity, executive dysfunction, and reward-processing abnormalities. Neuroimaging studies showed convergent alterations in dopaminergic reward pathways and prefrontal-limbic circuitry involved in craving and impaired inhibitory control. However, GD was more associated with online peer attachment and immersive digital engagement, whereas AUD was more associated with physiological dependence and neurotoxic effects. Both disorders were associated with impaired academic functioning, maladaptive coping and psychosocial dysfunction during adolescence.

**Discussion:**

Gaming Disorder (GD) and Alcohol Use Disorder (AUD) are distinct clinical but neurobehaviorally and developmentally vulnerable. Integrated prevention and intervention approaches targeting common transdiagnostic mechanisms may improve early identification and mental health outcomes among adolescents and young adults.

## Introduction

During adolescence and young adulthood, the emotional, cognitive and social systems are rapidly developing. At this stage of development, reward-related limbic circuits develop earlier than prefrontal cortical systems involved in executive regulation, impulse control and long-term decision-making, thus making adolescents and young adults more vulnerable to addictive and risk-taking behaviors (Arain et al., 2013). Against this background, problematic digital gaming and alcohol consumption have become increasingly prevalent behavioral health problems among adolescents and young adults. Recent epidemiological data suggest a dramatic increase in problematic gaming behaviors and alcohol use among adolescents and young adults worldwide. Meta-analytical estimates indicate the prevalence of Gaming Disorder and problematic gaming behaviors among adolescents is between 3% and 10%, with higher prevalence rates among males and competitive online gamers (Zhou et al., 2024). Alcohol use has also been reported to be highly prevalent among youth populations worldwide, with early initiation associated with increased risk of long-term substance dependence, cognitive impairment and psychosocial dysfunction (Farnia et al., 2024). Despite both behavioral and substance-related addictions being increasingly recognized among adolescents, prevention and intervention efforts often remain siloed between digital behavioral health and substance use frameworks.

Gaming Disorder (GD) was included in the International Classification of Diseases-11 (ICD-11) category “*Disorders Due to Addictive Behaviors*” by the World Health Organization (2019), which has increased the recognition of excessive gaming as a clinically significant behavioral addiction that shares several neurobehavioral features with substance-related disorders, such as Alcohol Use Disorder (AUD), while being diagnostically distinct (Reed et al., 2019). The ICD-11 framework emphasizes common mechanisms of impaired control, compulsive engagement, reward sensitivity, and persistence despite negative consequences, but does not equate behavioral addictions with substance-related addictions. Gaming Disorder in ICD-11 is characterized by impaired control over gaming, increasing priority given to gaming over other life activities, and persistence of gaming despite negative consequences, usually for at least 12 months. In contrast, Alcohol Use Disorder (AUD), as conceptualized in DSM-5 and ICD-based frameworks, is defined as problematic alcohol use associated with craving, impaired control, tolerance, withdrawal symptoms, and significant psychosocial or occupational dysfunction. Both conditions involve compulsive reinforcement-seeking and diminished self-regulation, but AUD also involves pharmacological dependence and direct neurophysiological toxicity, which clinically distinguishes it from behavioral addictions.

Emerging evidence suggests that both Gaming Disorder and Alcohol Use Disorder are defined by dysregulated reward processing, impaired executive functioning, increased cue-reactivity, maladaptive emotional regulation, and compulsive reinforcement-seeking behaviors (Brand et al., 2019; Volkow et al., 2019). Neuroimaging evidence also indicates convergent alterations in the nucleus accumbens, amygdala, anterior cingulate cortex, ventral striatum, and prefrontal cortical regions, suggesting overlapping neurocognitive vulnerabilities for craving, impulsivity, reward anticipation, and inhibitory control deficits. The Interaction of Person-Affect-Cognition-Execution (I-PACE) model by Brand et al. (2016, 2019) is one of the most influential contemporary models explaining the development and maintenance of behavioral addictions. The I-PACE model describes addictive behaviors as the result of dynamic interactions between predisposing personality traits, affective responses, cognitive biases and deficits in executive functioning. The model also proposes that repeated engagement in rewarding behaviors strengthens maladaptive reinforcement-learning mechanisms, cue-reactivity, craving, and impaired inhibitory control over time. Recent neurobiological extensions of the I-PACE model have implicated dysfunction within the ventral striatum, dorsolateral prefrontal cortex, anterior cingulate cortex, and salience-processing networks, linking emotional dysregulation and executive dysfunction to persistent addictive behaviors. This framework is particularly relevant during adolescence, when the ongoing development of executive control systems may heighten susceptibility to impulsive reward-seeking behaviors such as excessive gaming and hazardous alcohol use.

## Scientific research gap

While there is growing evidence that behavioral and substance-related addictions develop in young people, the literature has predominantly focused on Gaming Disorder and Alcohol Use Disorder as unique clinical problems. This siloed approach constrains our understanding of common vulnerabilities in development, psychosocial risk factors, and neurobehavioral mechanisms that may underlie the onset and persistence of both disorders in adolescence. Furthermore, few reviews have integrated evidence across emotional regulation, executive functioning, reward neurocircuitry, developmental psychopathology, and psychosocial outcomes in a cohesive developmental framework. This narrative review addresses this gap by conceptualizing problematic gaming and alcohol use as different addictive disorders that share neurobiological, cognitive, emotional and social pathways. Instead of treating the two disorders as one, this perspective highlights common developmental vulnerabilities and transdiagnostic reinforcement mechanisms that may come together during adolescence and young adulthood. By integrating evidence from neuroimaging, developmental neuroscience, psychosocial research and cognitive-behavioral models, the review strives to offer a multidimensional understanding of how behavioral and substance-related addictions may interact during key stages of brain development.

## Need for an integrative perspective

An integrated perspective is important because adolescents often engage in multiple reward-seeking behaviors at the same time, with overlapping vulnerabilities involving impulsivity, emotional dysregulation, sensation seeking, and impaired executive control. Although Gaming Disorder and Alcohol Use Disorder share common neurobehavioral mechanisms, important distinctions remain. Gaming problems are more closely related to immersive digital engagement and attachment to online peers, while Alcohol Use Disorder is associated with physiological dependence and neurotoxic effects. Emerging evidence also suggests gender-related differences, with male gender more likely to be associated with competitive gaming behaviors and female gender with more emotion-focused coping in substance use contexts (Zhou et al., 2024; Kuntsche et al., 2014). Greater understanding of these overlapping but distinct pathways may inform more balanced prevention and intervention efforts in clinical, educational, and public mental health contexts.

## Methodology

### Review design

This narrative review was conducted following the Scale for the Assessment of Narrative Review Articles (SANRA) framework that emphasizes scientific reasoning, transparency, methodological clarity, and balanced interpretation in narrative synthesis (Baethge et al., 2019). In contrast to systematic reviews, narrative reviews permit wider conceptual integration of heterogeneous evidence. Hence, this methodology is particularly appropriate for the comparative synthesis of neurobiological, psychological, and psychosocial dimensions of Gaming Disorder and Alcohol Use Disorder among adolescents and young adults.

### Search strategy

A structured literature search was conducted between January and March 2025 across PubMed, Scopus, PsycINFO, and Google Scholar for peer-reviewed studies published between 2010 and 2025 that examined neurobiological, psychological, cognitive, and psychosocial correlates of Gaming Disorder and Alcohol Use Disorder among adolescents and young adults. The search strategy combined controlled vocabulary and free-text terms using Boolean operators, such as “AND” and “OR” and included keywords related to gaming disorder, alcohol use disorder, adolescents, neurobiology, executive functioning, reward systems, emotional regulation, and psychosocial outcomes. Additional modifications to the search strategy were made for each individual database and manual searches of reference lists of eligible studies and relevant reviews were performed to increase sensitivity and find further articles. The search was limited to English language, peer reviewed human studies involving adolescents and young adults aged 10–25 years. Conference abstracts, editorials, dissertations, non-peer-reviewed reports, and studies without sufficient methodological information were excluded.

### Inclusion/Exclusion criteria

Studies were included if they examined Gaming Disorder, problematic gaming, Alcohol Use Disorder, hazardous alcohol use, or related addictive behaviors among adolescents and young adults aged 10–25 years. The studies included in the review examined neurobiological, cognitive, emotional, behavioral, psychosocial, or developmental correlates of addiction and used quantitative, qualitative, longitudinal, neuroimaging, experimental, cross-sectional or review methodologies. Only peer-reviewed articles published in English-language journals between 2010 and 2025 were included.

Studies were excluded if they focused exclusively on adult populations older than 25 years of age or if they examined psychiatric and neurological outcomes unrelated to addictive behaviors. Editorials, conference proceedings, protocols, commentaries, dissertations and non-peer-reviewed publications were also excluded. In addition, studies that lacked sufficient methodological clarity or outcome reporting, were not available in full-text format or published in languages other than English were not included in the review.

### Study screening

**FIGURE 1:**
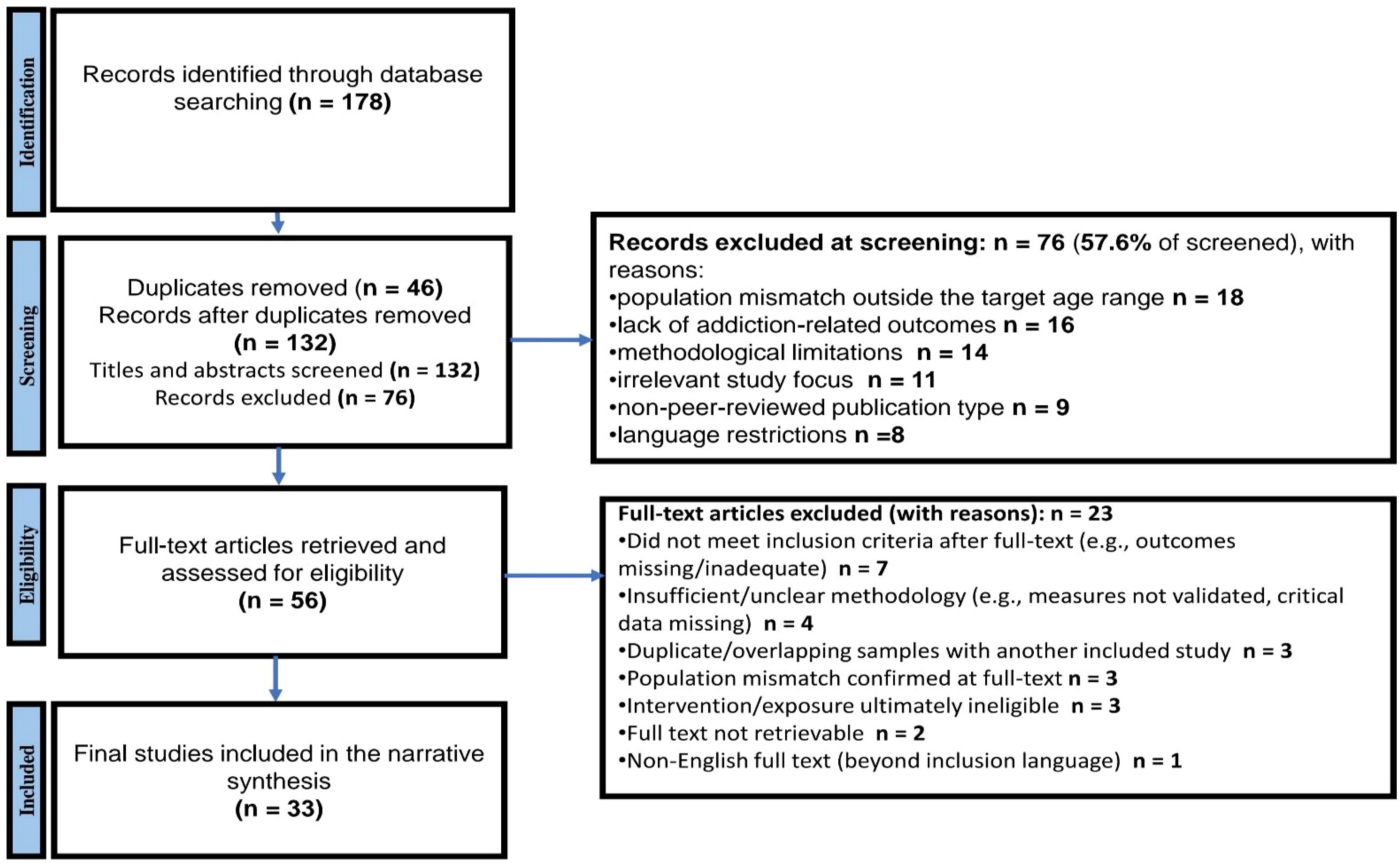
PRISMA flow diagram of study selection.

A total of 178 records were retrieved from database searching, from which 46 duplicates were removed. After screening titles and abstracts of 132 records, 56 full-text articles were assessed for eligibility. Twenty-three studies were excluded due to methodological and eligibility related concerns, thus 33 studies were included in the final narrative synthesis. Study screening was performed independently by two reviewers and disagreements were resolved by consensus. The study selection process is shown in Figure 1.

### Data extraction and thematic analysis

Systematic collection of information on study characteristics, participant demographics, addiction type, neurobiological findings, cognitive outcomes, emotional regulation patterns, and psychosocial correlates was performed during data extraction. After data extraction, a thematic synthesis approach was adopted to identify four overarching domains: (1) neurobiological similarities and distinctions, (2) emotional regulation and impulsivity, (3) cognitive and academic consequences, and (4) sociocultural vulnerabilities and resilience factors. For the sake of comparability, both convergent and divergent findings concerning Gaming Disorder and Alcohol Use Disorder were integrated to give a balanced and multidimensional account of shared and disorder-specific mechanisms.

### Quality appraisal

Quality assessment was performed following SANRA guided principles of narrative synthesis. The methodological rigor, developmental relevance, clarity of outcomes, sample size, and use of validated measures were assessed for each study. Studies using neuroimaging, longitudinal designs, or meta-analyses were given priority as they provide stronger evidence. Studies with insufficient or unclear methodology were excluded. Divergent findings were retained to ensure a balanced interpretation.

### Ethical considerations

This article is a narrative review based on the synthesis of previously published peer-reviewed studies. No primary data were collected or analyzed, and therefore ethical approval was not required. It was assumed that all studies included in this review followed relevant ethical standards as mentioned in the respective publication. The review process followed academic integrity and principles as described in the Declaration of Helsinki.

## Results

### Findings

The characteristics and key findings of the included studies are summarized in Table 1. The included studies showed a significant overlap between GD and AUD in terms of reward dysregulation, impulsivity, executive dysfunction, and emotional dysregulation. Neuroimaging data consistently demonstrated changes in prefrontal-limbic circuitry and dopaminergic reward pathways, supporting shared neurobehavioral vulnerabilities in behavioral and substance-related addictions.

**TABLE 1:** Characteristics and key findings of included studies examining Gaming Disorder and alcohol use disorder among adolescents and young adults (2010–2025).

No.	Author(s), Year	Study Design	Population / Age group	Primary focus	Key findings relevant to the review
1	Brand et al., 2019	Theoretical Model (I-PACE)	Adolescents	Behavioral addiction mechanisms	Proposed that addictive behaviors emerge through interactions among affective, cognitive, and executive dysfunction processes.
2	Wang et al., 2021	fMRI Study	15–22 years	Neural correlates of Gaming Disorder	Demonstrated hyperactivation of the nucleus accumbens and reduced prefrontal connectivity associated with impaired cognitive control.
3	Lees et al., 2020	Neuroimaging review	12–20 years	Alcohol-related brain changes	Reported gray- and white-matter alterations associated with executive dysfunction in adolescents exposed to alcohol.
4	Hamidullah et al., 2020	Longitudinal MRI study	13–18 years	Alcohol exposure and cognition	Chronic alcohol exposure was associated with reduced prefrontal cortical thickness and impaired learning efficiency.
5	Zhang et al., 2025	fMRI study	16–24 years	Functional connectivity in gaming	Identified disrupted prefrontal–anterior cingulate connectivity linked to poor inhibitory control.
6	Schultz, 2016	Neuroscience review	Adolescents	Dopamine reward processing	Suggested that addiction involves maladaptive reward prediction-error learning.
7	Volkow et al., 2019	Neurobiological review	Adolescents	Reward circuitry in addiction	Reported overlapping dopaminergic signatures across behavioral and substance-related addictions.
8	Burleigh et al., 2022	Meta-analysis	10–25 years	Cross-addiction vulnerability	Found significant co-occurrence between gaming-related problems and substance use behaviors.
9	Blasi et al., 2019	Cross-sectional study	12–19 years	Emotion regulation in gaming	Escapism, anxiety, and emotional dysregulation predicted problematic gaming behavior.
10	Kuntsche et al., 2014	Review	13–20 years	Drinking motives	Peer influence and stress-related coping emerged as major predictors of adolescent alcohol use.
11	Colder Carras et al., 2024	Policy review	Adolescents	Prevention strategies	School-based digital well-being interventions reduced gaming-related harm.
12	Poulton and Hester, 2020	Cross-addiction Survey	15–23 years	Reward sensitivity	Shared impulsivity and reward-seeking tendencies were observed across gaming and alcohol use.
13	Kräplin et al., 2021	Behavioral study	16–21 years	Cognitive control in gaming	Demonstrated impaired attention, inhibitory control, and decision-making speed among problematic gamers.
14	Mercurio et al., 2020	Developmental review	Adolescents	Adolescent brain maturation	Highlighted imbalance between limbic reward drive and immature executive control systems.
15	Heatherton and Wagner, 2011	Conceptual paper	Adolescents	Self-regulation failure	Proposed loss of self-regulation as a core mechanism underlying addictive behaviors.
16	Canham et al., 2016	Longitudinal survey	13–18 years	Social isolation and media use	Excessive media and alcohol use were associated with loneliness and social withdrawal.
17	Kuss and Lopez-Fernandez, 2016	Systematic review	Adolescents	Internet addiction treatment	Cognitive-behavioral therapy and mindfulness interventions showed therapeutic promise.
18	Krossbakken et al., 2018	Intervention trial	14–20 years	Parental monitoring	Structured parental monitoring reduced excessive gaming behaviors.
19	Schacht et al., 2013	fMRI study	15–18 years	Alcohol cue-reactivity	Anterior cingulate and amygdala activation predicted alcohol craving responses.
20	Luciana et al., 2018	Neurodevelopmental study	14–25 years	Adolescent substance exposure	Alcohol exposure altered reward-control balance during neurodevelopment.
21	Moshel et al., 2024	Review	Adolescents	Gaming neuropsychology	Reported deficits in attention and impulse inhibition among addictive behaviors.
22	Park et al., 2017	Neurocognitive study	13–19 years	Gaming cue-reactivity	Identified dopaminergic overactivation in response to gaming-related visual stimuli.
23	Zahr and Pfefferbaum, 2017	MRI study	14–18 years	Binge drinking effects	Alcohol exposure was associated with white-matter integrity loss.
24	Zhu et al., 2023	Cross-sectional study	17–23 years	Impulsivity in gaming	Problematic gamers demonstrated elevated impulsivity scores compared with controls.
25	Zheng et al., 2017	Longitudinal study	14–19 years	Predictors of drinking	Low inhibitory control predicted initiation of alcohol use.
26	Zhang et al., 2024	ERP study	16–22 years	Reward anticipation in gaming	Enhanced reward anticipation signals were identified among problematic gamers.
27	Spillane et al., 2012	Longitudinal study	14–17 years	Sensation seeking	High sensation seeking predicted addictive behavior initiation.
28	Boffo et al., 2019	Experimental Study	Adolescents	Cognitive bias modification	Bias modification reduced alcohol-related approach tendencies.
29	Koob and Volkow, 2010	Neurocircuitry Review	Adolescents	Addiction neurobiology	Described transition from impulsivity to compulsivity across addictions.
30	Mestre-Bach and Potenza, 2023	Neuroimaging Meta-analysis	10–25 years	Gaming and substance comparison	Shared striatal hyperactivity patterns were identified across addictions.
31	Hui and Gang, 2014	PET study	16–20 years	Dopamine depletion	Alcohol exposure reduced dopamine receptor binding within the striatum.
32	Amlung et al., 2017	Behavioral economics study	Adolescents	Delay discounting	Gamers and drinkers demonstrated steeper delay discounting patterns.
33	Carey et al., 2022	Narrative review	Adolescents	Gaming-related harm	Psychological harm associated with gaming mirrored features observed in substance dependence.

The comparative psychological, cognitive, and psychosocial features of Gaming Disorder (GD) and Alcohol Use Disorder (AUD) are presented in Table 2. Both Gaming Disorder and Alcohol Use Disorder were related to impaired emotional regulation, executive dysfunction, compulsive engagement and psychosocial impairment. However, Gaming Disorder was more strongly associated with online peer attachment and virtual identity reinforcement whereas Alcohol Use Disorder was more strongly associated with physiological dependence and interpersonal dysfunction.

**TABLE 2:** Comparative psychological, cognitive, and psychosocial features of Gaming Disorder and alcohol use disorder.

Domain	Gaming Disorder	Alcohol use disorder (AUD)	Shared / comparative interpretation	Supporting studies
Emotional regulation	Escapism, irritability, frustration intolerance, emotional dependence on gaming	Mood instability, guilt, emotional blunting, stress-related drinking	Both conditions involve maladaptive coping and emotional dysregulation	Blasi et al., 2019; Kuntsche et al., 2014
Cognitive functioning	Attention deficits, sleep disruption, reduced working memory	Impaired executive control, slowed processing, memory impairment	Both impair academic functioning and self-regulation	Kräplin et al., 2021; Hamidullah et al., 2020
Behavioral control	Compulsive gaming, craving, impulsive engagement	Risk-taking, compulsive drinking, impaired inhibitory control	Shared impulsivity and reward-seeking tendencies	Zhu et al., 2023; Zheng et al., 2017
Social functioning	Online peer attachment, virtual identity reinforcement, social withdrawal	Peer pressure, interpersonal conflict, relational dysfunction	Both disrupt interpersonal functioning, although gaming often involves distorted virtual connectedness rather than complete isolation	Canham et al., 2016; Poulton and Hester, 2020
Identity and self-concept	Dependence on digital achievement and online validation	Substance-centered identity formation and reduced autonomy	Fragile self-concept and delayed psychosocial maturation	Heatherton and Wagner, 2011
Long-Term Consequences	Behavioral addiction, cross-addiction vulnerability	Chronic dependency, neurotoxicity, psychosocial impairment	Shared reinforcement dysregulation with differing physiological impacts	Burleigh et al., 2022; Koob and Volkow, 2010

The comparative neurobiological characteristics of Gaming Disorder (GD) and Alcohol Use Disorder (AUD) are presented in Table 3. Both disorders exhibited reward-processing and executive-control systems abnormalities, such as dopaminergic dysregulation and impaired inhibitory control. Gaming Disorder was mostly associated with functional connectivity changes, whereas Alcohol Use Disorder was more related to structural neurotoxicity in prefrontal and hippocampal regions.

**TABLE 3:** Comparative neurobiological characteristics of Gaming Disorder and alcohol use disorder.

Neurobiological domain	Gaming Disorder	Alcohol use disorder (AUD)	Comparative interpretation	Supporting studies
Reward system activity	Hyperactivation of mesolimbic reward pathways and cue-reactivity	Dopamine release followed by depletion and impaired reward prediction	Both disorders involve reward dysregulation driving compulsive engagement	Wang et al., 2021; Volkow et al., 2019; Schacht et al., 2013
Executive control	Functional underconnectivity between prefrontal and limbic regions	Structural and metabolic damage within prefrontal cortical regions	Gaming is associated primarily with functional impairment, whereas alcohol causes structural neurotoxicity	Zhang et al., 2025; Zahr and Pfefferbaum, 2017
Memory and learning	Altered hippocampal activation without major volumetric loss	Hippocampal shrinkage and impaired neurogenesis	Alcohol demonstrates greater neurotoxicity in memory-related systems	Luciana et al., 2018; Lees et al., 2020
Neurotransmitter regulation	Elevated dopamine and glutamate reactivity with reduced inhibitory control	Dysregulation of dopamine, serotonin, GABA, and glutamate systems	Both demonstrate excitatory–inhibitory imbalance	Hui and Gang, 2014; Koob and Volkow, 2010
Developmental vulnerability	Delayed prefrontal maturation and reward hypersensitivity	Early alcohol exposure linked to long-term cognitive deficits	Adolescence represents a critical neurodevelopmental vulnerability window for both disorders	Mercurio et al., 2020; Hamidullah et al., 2020
Cue-reactivity and craving	Gaming-related visual cue overactivation	Alcohol-related cue-triggered craving activation	Both conditions demonstrate heightened salience attribution and craving-related neural responses	Park et al., 2017; Schacht et al., 2013

## Discussion

This narrative review focuses on the increasing overlap of Gaming Disorder (GD) and Alcohol Use Disorder (AUD) in adolescents and young adults with both apparently sharing similar neurobehavioral vulnerabilities. Although GD is classified as a behavioral addiction and AUD a substance use disorder, the two disorders overlap in terms of common neurological, cognitive and affective pathways related to reward processes, impulsivity and deficits in self-regulation. These disorders are highly associated with emotional dysregulation, inadequate coping and increased impulsivity at the psychological level. For example, escapism and anxiety are important factors in problematic gaming behaviors (Blasi et al., 2019) and peer influence and stress-related coping are important predictors of alcohol use in adolescents (Kuntsche et al., 2014). These findings are consistent with the broader developmental literature suggesting that externalizing coping strategies during adolescence may lead to maladaptive behavioral patterns (Perzow et al., 2021). In addition, cognitive deficits in attention, working memory, and decision-making may contribute to addictive engagement and further impair academic performance and self-regulation (Kräplin et al., 2021; Hamidullah et al., 2020).

Neurobiologically the findings suggest significant overlap in reward circuit failure in both illnesses. Functional neuroimaging studies suggested that GD is related with hyperactivation of mesolimbic dopaminergic pathways, especially in the nucleus accumbens (NAc) and ventral tegmental area (VTA) (Wang et al., 2021). Likewise, alcohol exposure leads to dopamine release and subsequent dopamine depletion that disrupts reward prediction and promotes substance-seeking behavior (Volkow et al., 2019). The enhanced cue and desire responses in both situations could be related to the changed processing of rewards (Schacht et al., 2013).

Nevertheless, large neurobiological differences continue to be evident. Whereas GD has been primarily associated with functional deficits of the prefrontal-limbic connectivity, AUD has been associated with structural and metabolic damage of the prefrontal and hippocampal regions (Zhang et al., 2024; Zahr and Pfefferbaum, 2017). As such, gaming-related neuroplastic changes may be more reversible through behavior modification. By contrast, alcohol-related neurotoxicity may have more lasting effects on cognition and behavior (Luciana et al., 2018). Vulnerability to gaming and alcohol use behaviors From developmental and psychosocial points of view, adolescence is a period of identity development, increased reward sensitivity and vulnerability to peer influence, all of which contribute to vulnerability. Loneliness, peer conformity and social reinforcement have been found in longitudinal studies to be associated with problematic gaming and alcohol use, which lead to negative psychosocial developmental and interpersonal functioning outcomes (Canham et al., 2016; Poulton and Hester, 2020).

The co-occurrence of gaming disorders and alcohol use behaviors suggests a need for integrated preventative interventions. Impulsivity, sensory seeking and reward sensitivity are among shared pathways across both conditions (Burleigh et al., 2022; Mestre-Bach and Potenza, 2023). These findings are in line with the Interaction of Person-Affect-Cognition-Execution model (I-PACE), which posits those addictive behaviors stem from the interaction of personal predispositions, affective states, cognitive biases, and executive dysfunction (Brand et al., 2016). Our data suggest that preventative and intervention programs for GD and AUD may fail to identify important shared vulnerabilities when they focus on the two conditions separately. Therefore, common cognitive systems such as emotional regulation, reward processing, or executive functioning may have more general clinical implications. Cognitive-behavioral therapy, mindfulness-based interventions, and school-based prevention programs, adapted to developmental and contextual needs, are promising strategies to tackle both behavioral and substance-related addictive behaviors (Kuss and Lopez-Fernandez, 2016; Colder Carras et al., 2024).

## Implications of the study

The findings of this review indicate substantial overlap of Gaming Disorder (GD) and Alcohol Use Disorder (AUD) in terms of emotional regulation, impulsivity, executive dysfunction and reward-processing mechanisms in adolescents and young adults. These common neurobehavioral vulnerabilities justify the need for integrated prevention and intervention frameworks for behavioral addictions and substance use disorders to be simultaneously targeted. School-based mental health programs, family-based interventions, cognitive-behavioral therapy (CBT), mindfulness-based approaches and emotional regulation training may be particularly useful when adapted to target common transdiagnostic mechanisms. Early identification in adolescence, a developmental period characterized by increased neuroplasticity and reward sensitivity, may improve long-term psychosocial and cognitive outcomes.

## Limitations of the study

Several limitations should be considered when interpreting the findings of this review. The narrative review methodology provides the advantage of wide conceptual synthesis but lacks the same methodological rigor and quantitative precision as systematic reviews and meta-analyses. The heterogeneity in study design, assessment tools, populations, and outcome measures may have hindered the direct comparison of the included studies. A significant portion of the evidence included was based on cross-sectional and short-term studies, which limits our understanding of causality and inferences on long-term development. The inclusion of selected conceptual and review papers, although critical for theoretical integration, may have led to interpretive subjectivity. The review protocol was not registered in PROSPERO or OSF, which could influence the methodological transparency and reproducibility of the review.

## Future directions

Future research should include longitudinal, neuroimaging and developmental studies to better understand the long-term neurocognitive consequences of early gaming and alcohol exposure in adolescence. More attention should also be paid to gender-specific pathways, reward sensitivity, emotional regulation and sociocultural influences such as peer interactions, digital environments and online social reinforcement. Further examination of cross-addiction vulnerability and transdiagnostic mechanisms may contribute to the development of integrated intervention frameworks that address multiple addictive behaviors concurrently. Furthermore, culturally appropriate prevention strategies and technology-based interventions should be explored in educational and public mental health settings.

## Conclusion

This review highlights the important emotional, cognitive, psychosocial and neurobiological vulnerabilities between GD and AUD, particularly in relation to reward dysregulation, impulsivity and executive dysfunctioning. Although both disorders remain clinically distinct, their overlapping developmental and neurobehavioral mechanisms support the call for integrated prevention and intervention approaches. Identifying these shared vulnerabilities during adolescence may enable earlier identification, targeted intervention and improved long-term psychosocial and mental health outcomes.

## Data Availability

The original contributions presented in this study are included in this article/supplementary material, further inquiries can be directed to the corresponding author.
